# Effect of HIV/HCV Co-Infection on the Protease Evolution of HIV-1B: A Pilot Study in a Pediatric Population

**DOI:** 10.1038/s41598-018-19312-2

**Published:** 2018-02-05

**Authors:** Sara Domínguez-Rodríguez, Patricia Rojas, Carolina Fernández McPhee, Israel Pagán, María Luisa Navarro, José Tomás Ramos, África Holguín

**Affiliations:** 1 HIV-1 Molecular Epidemiology Laboratory, Microbiology and Parasitology Department, Hospital Ramón y Cajal-IRYCIS and CIBER-ESP, Madrid, 28034 Spain; 2 Department of Pediatric Infectious Diseases, Hospital Universitario Gregorio Marañón-IisGM-UCM-RITIP-CoRISPe, Madrid, 28009 Spain; 3 Centro de Biotecnología y Genómica de Plantas (UPM-INIA), Campus Montegancedo, Pozuelo de Alarcón, 28223 Madrid, Spain; 4 Pediatric Department, Hospital Clínico Universitario and Universidad Complutense, Madrid, 28040 Spain

## Abstract

This pilot study evaluates in pediatric patients the impact of HIV/HCV coinfection in the molecular evolution of the HIV-1 subtype B protease (HIV-1BPR). For this study, HIV-1B/HCV coinfected (15) and HIV-1B monoinfected (56) patients with available HIV-1B *pol* sequences were enrolled. Both groups of patients had comparable gender frequencies and average age, time of infection, antiretroviral treatment (ART) exposure and time under ART. Prevalence of drug resistance mutations (DRM), genetic diversity, number of synonymous (*d*_S_) and non-synonymous (*d*_N_) mutations per site and selection pressures (*d*_N_ − *d*_S_) in the HIV-1BPR were estimated and compared between mono- and coinfected patients. Both HIV-1B populations presented similar genetic diversity (0.050 ± 0.02 *vs*. 0.045 ± 0.01) and *d*_S_ (0.074 ± 0.03 *vs*. 0.078 ± 0.04). In turn, in coinfected patients the HIV-1BPR had higher *d*_N_ (0.045 ± 0.01 *vs*. 0.024 ± 0.01) and *d*_N_-*d*_S_ (−0.026 ± 0.02 *vs*. −0.048 ± 0.04) values, and less amino acid sites under purifying selection (4.2% *vs*. 42.1%) than in monoinfected patients. Accordingly, in co-infection with HCV, the HIV-1BPR sites 50, 53, 82, 84 and 88 - associated with resistance to PIs - were under neutral evolution, whereas these sites were under purifying selection in monoinfected patients. This pilot study suggests that HIV-1B may evolve differently in the presence than in the absence of HCV.

## Introduction

Coinfection by multiple pathogen genotypes/species is an important driver of disease progression^1^, which may have far-reaching consequences for within-host infection dynamics^2,3^. In 2016, approximately 2.3 million people living with HIV-1 were coinfected with Hepatitis C virus (HCV) worldwide^4^, and this coinfection is known to cause long-term complications in HIV-infected patients^5^. For instance, in children as well as in adults HIV-1/HCV coinfection accelerates the progression hepatic disease^6–8^. Although researchers agree on the impact of HIV-1 on HCV-associated disease, the influence of HCV on HIV-1 disease progression is still controversial, particularly in pediatric patients^9,10^. While some studies in HIV-1/HCV coinfected children reported slower disease progression than in monoinfected patients^11^, other works showed similar progression rates in both groups of patients^6,7,12^. HIV-1 disease progression has been associated with the level of genetic diversity in the virus population^13^. Thus, understanding how HIV-1/HCV coinfection affects HIV-1 population genetic diversity may contribute to improve the clinical management of HIV-1 infections: Identifying sites under diversifying selection offers information about the mechanisms by which HIV-1 achieves immunological escape or drug resistance, and the characterization of sites under purifying selection may provide relatively immutable targets to design more efficient treatments and diagnostic methods^14^.

Various clinical factors have been associated with the level of genetic diversity in HIV-1 populations and with selection pressures in the virus genome. Genetic diversity increases with lower levels of CD4 count^15^, higher viral load^16,17^, larger virus exposure times^18^, age, and with different ART regimens^19,20^. In addition, a recent study showed that in adult patients, coinfection with HCV or Hepatitis B virus was an important predictor of the appearance of drug resistance mutations (DRM) and of *d*_*S*_ values in the HIV-1 genome^21^. However, in children little is known about HIV-1 evolution in the presence of HCV.

In this pilot study, we provide, for the first time, data on how HCV infection may affect HIV-1 evolution in pediatric patients by analyzing the prevalence of drug resistance mutations (DRM), the genetic diversity, the number of synonymous and non-synonymous mutations per site and the selection pressures in the HIV-1B protease of virus isolates in single infection and in coinfection with HCV.

## Materials and Methods

### Study population

For this pilot study, we used data from the Madrid cohort of HIV-infected children and adolescents that has been extensively described in previous studies (ref.^22^ and Supplementary Information). Fifteen HIV-1B/HCV coinfected patients with available *pol* HIV-1 sequences collected between January 1993 and December 2015 were selected, as well as a control group of 56 HIV-1B monoinfected patients (Table S1). All 71 enrolled patients were Spanish and both groups had similar distributions of gender, age and time of HIV-1 infection, as the latter two factors have been previously associated with virus evolution^23^. With the aim of avoiding intrinsic subtype evolution features^24^ only patients carrying HIV-1 subtype B *pol* sequences were enrolled in the study. When more than one sequence was available, we selected the sequence that was collected closest to the date-of-birth. Patients with other known viral coinfections were excluded from this study. Importantly, none of the coinfected patients were treated for HCV at sampling time.

The project was approved by the Human Subjects Review Committee at University Hospital Ramón y Cajal (Madrid, Spain), and informed consent of the parents or guardians was obtained. All methods were carried out in accordance with relevant guidelines and regulations.

### Sequence data

We based our pilot study in the HIV-1B protease (HIV-1BPR) region since it has been shown to be suitable for analyses of HIV-1 adaptability due to its high variability and evolvability^25,26^. PR and retrotranscriptase (RT) *pol* sequences were retrieved from retrospective clinical routine antiretroviral resistance testing or from previous research studies published by our group^27–29^. HIV-1BPR sequence alignments were constructed using CLUSTALX v2.1^30^, and manually edited according to the amino acid (aa) sequences using MEGA v7^31^. The GenBank accession numbers of the sequences utilized in this pilot study are shown in Table S2.

### Drug resistance mutations

The acquired drug-resistance mutations (DRM) in pretreated patients at PR and RT were defined following the International AIDS Society–USA (IAS) 2017 list^32^. Among drug-naïve patients, transmitted DRM (TDR) were defined according to the mutation list as recommended by the WHO^33^ and using the Calibrated Population Resistance tool^34^.

### Detection of recombination

Previous phylogenetic analyses indicated that the utilized sequences clustered with those of representative isolates of HIV-1 subtype B^26–29^, and none of them clustered with any of the 90 circulating recombinant forms (CRF), including the 43 CRFs with subtype B sequences at *pol*. This was confirmed by analyzing recombination breakpoints in our HIV-1BPR alignment using the RDP4 package employing the default parameters^35^. Recombination signals detected with *P* < 0.05 by at least four different methods were considered as positive. Recombination breakpoints were not detected in any of the analyzed HIV-1BPR sequences (*P* > 0.784).

### Estimation of genetic diversity and selection pressures

To estimate evolutionary parameters, we only analyzed pediatric patients under ART that were vertically infected so that we avoided HIV-1/HCV superinfections. Pairwise genetic distances (*d*) were estimated using Tamura-3-parameters (T92) nucleotide substitution model as implemented in R Ape package^36,37^, which was the best-fitted nucleotide substitution model as determined by jModelTest 2.1.8^38^. Standard errors (SE) of each measure were based on 1,000 bootstrap replicates. Genetic diversity values were obtained using 11 and 41 HIV-1BPR sequences from co- and monoinfected patients, respectively, after removing outlier sequences with abnormally large pairwise genetic distances in order to avoid possible biases.

Selection pressures were calculated as the difference between the mean number of non-synonymous (*d*_*N*_) and synonymous (*d*_*S*_*)* nucleotide substitutions per site (*d*_*N*_ − *d*_*S*_) by Pamilo Bianchi method^39^ implemented in MEGA v7^31^ and by Li-Wu-and-Luo method implemented in R SeqInr package^40^. Since the two methods led to the same conclusions, only Pamilo-Bianchi results are shown. Individual values of *d*_*N*_ and *d*_*S*_ were also obtained. Tests for positive selection on individual codons were conducted using the fast unconstrained Bayesian approximation (FUBAR) method as in the Datamonkey server^41^. In all cases, *d*_*N*_ − *d*_*S*_ estimates were based on input neighbor joining trees inferred using the MG94 nucleotide substitution model. In order to avoid false-positives, only sites with FUBAR posterior probability of >0.90 were accepted as candidates for being under diversifying selection.

### Statistical analysis

Differences in patient and mutation frequencies between mono- and coinfected groups were analyzed by Fisher’s exact test with alpha = 0.05. Continuous variables were compared by Student’s T test for parametric variables, and by Wilcoxon rank test for non-parametric variables. Differences in genetic distances and evolutionary parameters were measured comparing 95% confidence intervals (CI95): values were not considered as significantly different when the mean value of a given parameter in one group was included in the CI95 of the value for the same parameter in the other group, as this indicated that both values were drawn from the same distribution. Multivariable logistic regression analysis was performed to explore the relationship between coinfection and the number of DRM to nucleoside reverse transcriptase inhibitors (NRTI). Codon *d*_*N*_ *−* *d*_*S*_ rank position and correlation codon analysis were carried out using the Spearman’s rank correlation analysis method wrapped in spearman package^42^, given that these variables were non-parametric. All statistical analyses and plots were carried out using R software^36^.

## Results

### Prevalence of HIV-1 transmitted and acquired drug resistance mutations in mono- and coinfected patients

We observed a similar proportion of sequences carrying DRM to NRTI (83.3% *vs*. 87.5%; *P* = 1.000), to NNRTI (30.3% *vs*. 48.8%, *P* = 0.510) at HIV-1BRT, and to PI at HIV-1BPR (33.3% *vs*. 50%; *P* = 0.750) in ART-treated mono and coinfected children (Figure S1 and Table S3). In agreement with this results, a multivariate lineal regression analysis adjusted by experience and time under NRTI monotherapy, HAART experience and viral load, revealed that HCV coinfection was not a risk factor for the development of DRM to NRTI (OR = 0.26, *P* = 0.269).

### HIV-1B population genetic diversity in mono- and coinfected patients

**Figure 1:**
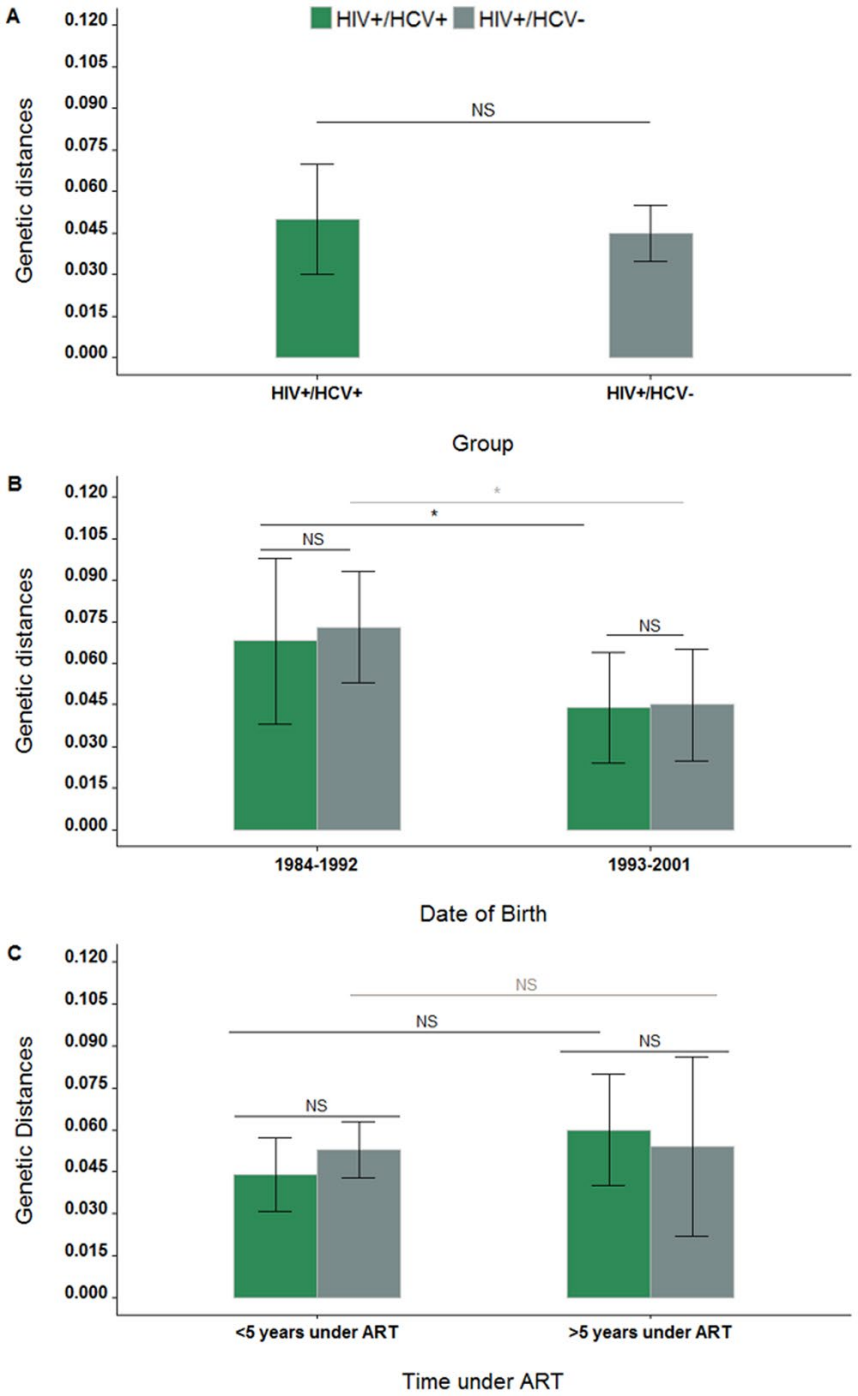
Genetic distances in monoinfected (grey) and coinfected (green) HIV-1BPR sequences. Panel A, Mean genetic distances of HIV-1BPR in coinfected (n = 11) and monoinfected (n = 41) pediatric treated patients. Panel B, Mean genetic distances of HIV-1B PR from both groups of patients according to date of birth period; P1: 1984-1992 (HIV/HCV n = 5, HIV n = 22); P2: 1993-2001 (HIV/HCV n = 6, HIV n = 22). Panel C, mean genetic distances according to time under ART considering around five years under ART. NS, not significant; *significant. Three outliers sequences from the coinfected group and seven from the monoinfected group were removed to avoid estimation biases.

Similar values of genetic diversity were observed in HIV-1B populations of mono- and coinfected children, either when these were analyzed as a whole (0.050 ± 0.020 *vs*. 0.045 ± 0.010) (Fig. 1A), categorizing sequences by birth date (0.073 ± 0.02 *vs*. 0.068 ± 0.03, for patients born between 1984-992; and 0.045 ± 0.02 *vs*. 0.044 ± 0.02, for patients born between 1993-2001) (Fig. 1B), or by time under ART (Fig. 1C). In addition, the genetic diversity of virus populations in patients born between 1984 and 1992 was higher than that of patients born between 1993 and 2001 regardless these were mono- or coinfected (Fig. 1B). When monoinfected children with more or less than 5 years under ART were compared, no differences in HIV-1BPR genetic diversity were observed, and the same result was obtained for coinfected children (Fig. 1C).

To rule out that the observed results were due to the sample size differences, we bootstrapped (10 times) the 41 sequences from the monoinfected group in ten groups of 11 sequences (sample size of co-infected patients). Average values of these randomized data sets did not significantly differ from the data derived from the 41 sequences (Table S4).

### Selection pressures (252)

**Figure 2:**
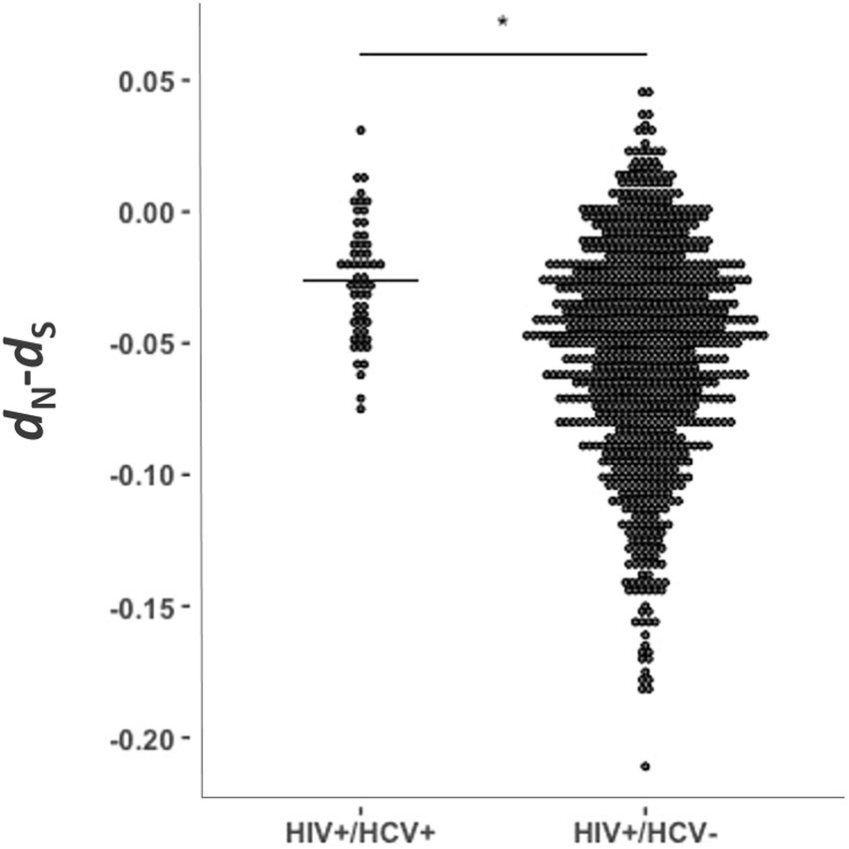
Selection pressures (*d*_*N*_ − *d*_*S*_) in the HIV-1BPR of coinfected and monoinfected patients. Selection pressures were estimated from HIV-1B PR sequences of 11 coinfected and 41 monoinfected treated children. Asterisks indicate significant differences between mono- and coinfected patients.

Selection pressures in the HIV-1BPR were assessed estimating *d*_*N*_ *− d*_*S*_. Also, *d*_*N*_ and *d*_*S*_ were estimated individually. The *d*_*S*_ values were similar in the HIV-1BPR of both mono- and coinfected patients (0.074 ± 0.03 *vs*. 0.078 ± 0.04, respectively), whereas virus *d*_*N*_ was higher in mono- than in coinfected patients (0.045 ± 0.01 *vs*. 0.024 ± 0.01). The *d*_*S*_ values were always higher than the corresponding *d*_*N*_ ones. Consequently, the HIV-BPR in the two groups of patients was under purifying selection, this being weaker in the co- than in monoinfected children (*d*_*N*_ *− d*_*S*_: −0.026 ± 0.002 *vs*. −0.048 ± 0.004, respectively) (Fig. 2).

**Figure 3:**
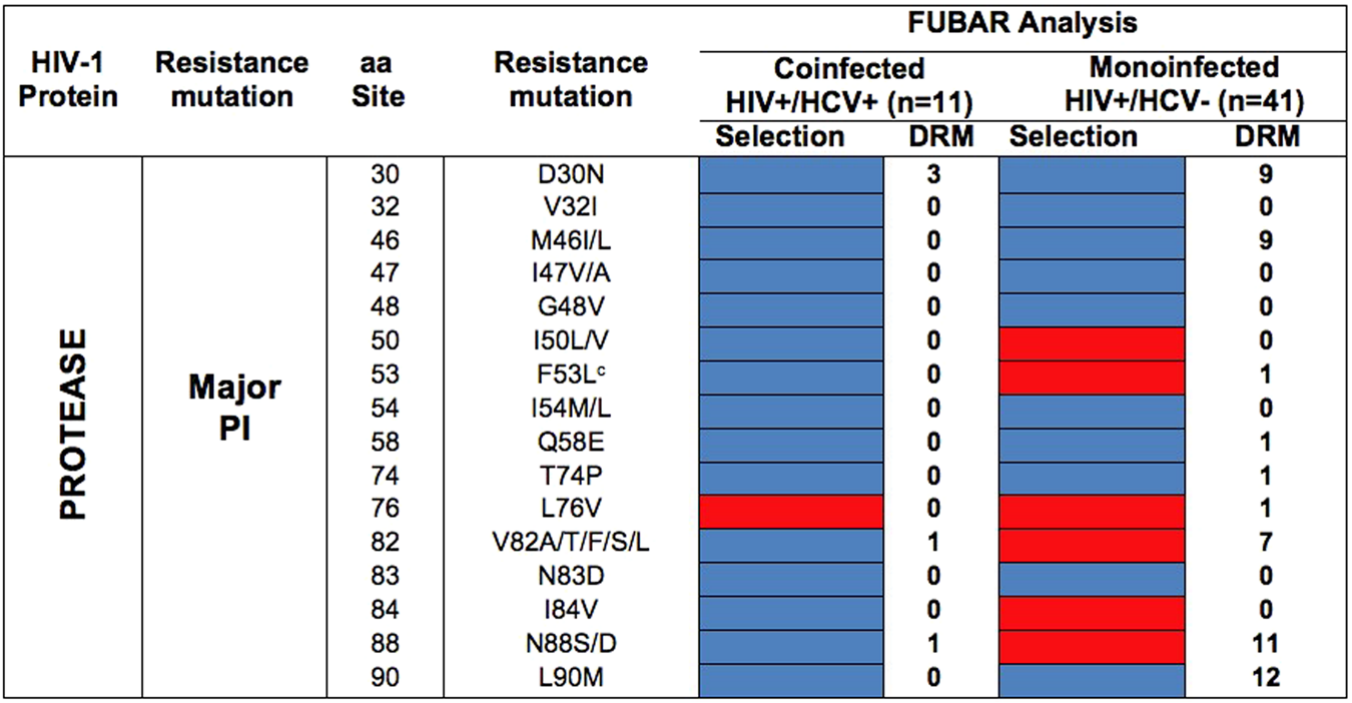
Selection pressures in the HIV-1BPR sites associated with major PI resistance in mono- and coinfected children. Red squares denote sites under purifying or negative selection, and blue squares denote sites under neutral evolution. PI, protease inhibitors; aa, amino acid site.

The frequency of sites under purifying selection in the HIV-1BPR was significantly lower in coinfected than in monoinfected children (4.2% *vs*. 42.1%; *χ*^*2*^ = 27.67, *P* = 3.2·10^−6^), with only a few (if any) sites under diversifying selection (0% *vs*. 2.1%; *χ*^*2*^ = 0.51, *P* = 0.47). When only the 16 PR amino acid sites associated with major PI resistance were analyzed, none was detected to be under diversifying selection neither in mono- nor in coinfected patients. Indeed, most of these sites were under neutral evolution in both virus populations (Fig. 3). However, selection pressures differed between mono- and coinfected children in 5 of these sites (amino acid sites 50, 53, 82, 84 and 88), which were under neutral evolution in the PR of HIV-1B isolates coinfected with HCV, and under purifying selection when HIV-1B was in monoinfection (Fig. 3). No correlation was found between the *d*_*N*_ *− d*_*S*_ values in the two virus populations under study at HIV-1BPR codons (*ρ* = 0.08, *P* = 0.425) (Figure S2).

We tested whether sample size differences between mono- and coinfected patients affected our estimates of selection pressures following the same approach as for genetic diversity. Again, average value of randomized data sets did not significantly differ from the data derived from the 41 sequences (Table S4).

## Discussion

HIV-1/HCV co-infection may have far-reaching consequences for AIDS progression in children^9,10^. However, very little is known on how this coinfection affects HIV-1 evolution. This pilot study addresses this subject by using one of the largest and best-characterized pediatric cohorts worldwide^43^, which provides a unique opportunity to analyze viral evolutionary parameters in the presence/absence of HCV in a patient population for which infection set-point and time of infection are known^6^.

We present tentative evidence indicating that HIV-1BPR from coinfected children had, higher *d*_N_ and, accordingly, higher *d*_*N*_ *− d*_*S*_ than HIV-1BPR from monoinfected children. This was associated with a significantly lower number of HIV-1BPR amino acid sites under purifying selection in co-infected patients as compared to HIV-1B monoinfected children. HIV-1PR is a robust enzyme that has a high tolerance to conformational changes. This allows HIV-1PR to rapidly acquire mutations that may lead to PI resistance^32^. In our study, the relaxed selection pressures detected in the HIV-1PR of coinfected patients suggest that this tolerance is more efficient when HCV and HIV-1 coexist. Indeed, five HIV-1PR sites where major DRM to PI appear were under neutral evolution in coinfected children, and under purifying selection in monoinfected children. These sites were located either in the PR flap region (codons 50 and 53) or in the substrate union site (82, 84, and 88), where non-synonymous nucleotide changes leading to the appearance of DRM to PI increase viral fitness^44^. This is in agreement with previous analyses showing that tolerance to mutational load associated with neutral evolution may allow a viral population to explore a wider range of mutations that can increase viral fitness^24,44^. Therefore, our results suggest that relaxed selection pressures in the HIV-1BPR upon coinfection with HCV may promote HIV-1 adaptation. We can only hypothesize on which mechanisms may drive this adaptation process: (i) competition with HCV may provide a different replicative environment, which could force HIV-1 to evolve higher fitness in this environment to efficiently compete with HCV^25^, or (ii) HCV proteins could complement the HIV-1BPR function, reducing PR structural constraints and favoring the appearance of amino acid changes^45^.

Further studies are needed to elucidate the mechanisms underlying the evolutionary patterns observed here. Interestingly, the higher values of *d*_*N*_ and *d*_*N*_ *− d*_*S*_ in co- than in monoinfected HIV-1B populations did not affected virus genetic diversity. This could be explained by the much higher weight of *d*_S_ mutations, that did not varied between mono- and coinfected HIV-1BPRs, in the viral genome as compared with *d*_N_ mutations. This would be in accordance with observations in other RNA viruses^46^, indicating that the lack of variation in genetic diversity levels between virus populations does not necessarily preclude the appearance of mutations that may potentially affect virus fitness^47^.

Some cautionary comment should be called upon our results. First, the number of coinfected patients that could be included in our analyses was small due to the low prevalence of HIV-1/HCV coinfection in the study cohort (6.2%) and to reduced sequences/samples availability^48,49^. However, reduction of the larger sample size of monoinfected patients to the same number of available HIV-1B sequences from coinfected patients did not alter our results. This suggests that the small sample size is sufficiently representative for a meaningful analysis, but studies in larger data sets are needed to test the generality of our results. Second, host immune system is likely to be impaired in mono- and coinfected children^50,51^. To minimize the effect of this impairment in our analyses, we only included coinfected and monoinfected children with a high rate of T CD4+ lymphocyte at sampling time. However, we cannot discard that each host could handle the two viral infections differently, exerting different immune pressure against them^51^. Moreover, the presence of both infections limits the ability of the innate immune system to fight infection in coinfected patients, even with similar T CD4+ rates than monoinfected^52^. Therefore, the observed differences between HIV-1B evolution in mono and coinfected children could be at least in part due to the differential selection pressures exerted by the patient’s immune system. Third, it has been reported that HIV-associated immunosuppression can increase *d*_*N*_/*d*_*S*_ ratios in some HCV proteins but not in others^53,54^. Thus, we cannot exclude that the observed effect of HCV coinfection in *d*_*N*_ − *d*_*S*_ can differ across HIV-1B proteins. Finally, differences in ART regimens experienced by each patient may affect HIV-1B evolution^55^. Given the constant appearance and implementation of new drugs and ART regimens since 1993 in the analyzed cohort, very few patients shared common ART regime histories. Thus, the assessment of the effect of ART regime in our analyses was not possible. Further analyses are needed to understand the potential effect ART experience in the presented results.

With the above-mentioned caveats, our findings contribute to understand the effect of HCV co-infection on HIV-1 evolution, and highlight the relevant role that virus-virus interactions may have in determining the outcome of infection.

## Electronic supplementary material

Supplementary Information
