# Supplementary material for: Effect of HIV/HCV Co-Infection on the Protease Evolution of HIV-1B: A Pilot Study in a Pediatric Population
Source: Sci Rep. 2018 Feb 5;8:2347. doi: 10.1038/s41598-018-19312-2 (PMC5799169; doi:10.1038/s41598-018-19312-2)
Supplement: Supplementary file 1 — Supplementary Information [file 41598_2018_19312_MOESM1_ESM.pdf]

Supplementary Information Manuscript SREP-17-35879B

EFFECT OF HIV/HCV CO-INFECTION ON THE PROTEASE EVOLUTION OF HIV-1B INFECTING PEDIATRIC POPULATIONS

Sara Domínguez Rodríguez[1], Patricia Rojas Sánchez[1], Carolina Fernández Mcphee[2], Israel Pagán[3], María Luisa Navarro[2], José Tomás Ramos[4] and África Holguín*[1].

[1] HIV-1 Molecular Epidemiology Laboratory, Microbiology and Parasitology Department, Hospital Ramón y Cajal-IRYCIS and CIBER-ESP, Madrid (28034), Spain; sara.dominguez.r@gmail.com,

[2] Department of Pediatric Infectious Diseases, Hospital Unviersitario Gregorio Marañón-IisGM-UCM-RITIP-CoRISPe, Madrid (28009), Spain; *carolina.fernandezmcphee@gmail.com*, *marisa.navarro.gomez@gmail.com*

[3] Centro de Biotecnología y Genómica de Plantas (UPM-INIA), Campus Montegancedo, Pozuelo de Alarcón (28223), Madrid Spain; *jesusisrael.pagan@gmail.com*

[4] Pediatric Department, Hospital Clínico Universitario and Universidad Complutense, Madrid (28040), Spain; *josetomas.ramos@salud.madrid.org*

* Correspondence: *africa.holguin@salud.madrid.org*

NOTE: This research has been done within the framework of CoRISpe cohort (Pediatric Spanish National Network) of the Spanish National AIDS Network (RIS, RD12/0017). Participants acknowledged included in this consortium, excluding authors, are listed below:

[2]Hospital Gregorio Marañón, Madrid (Spain): María Luisa Navarro, Jesús Saavedra, Mar Santos, Mª Angeles Muñoz, Beatriz Ruiz, Carolina Fernandez Mc Phee, Santiago Jimenez de Ory, Susana Alvarez

[4]Hospital Clínico San Carlos, Madrid (Spain): José Tomás Ramos, Isabel Cuellar

[5]Hospital La Paz, Madrid (Spain): María José Mellado, Luis Escosa, Milagros García Hortelano, Talía Sainz

[6]Hospital Doce de Octubre, Madrid (Spain): María Isabel González- Tomé, Pablo Rojo, Daniel Blázquez, Elisa Fernández, Berta Zamora, Cristina Garcia-Navarro, Manuela Martin, Carlos Velo, Ana Martinez de Aragon

[7]Hospital de Getafe, Getafe (Spain): Luis Prieto, Sara Guillén

[8]Hospital Universitario de Móstoles, Móstoles (Spain): Miguel Ángel Roa

[9]Hospital Príncipe de Asturias, Alcalá de Henares (Spain): José Beceiro

[10]Hospital Niño Jesús, Madrid (Spain): Jorge Martínez

[11]Hospital de Torrejón, Torrejón (Spain): Katie Badillo

[12]Hospital de Donostia, San Sebastián (Spain): Miren Apilanez

[13]Hospital de Cruces, Bilbao: Itziar Pocheville

[14]Hospital de Basurto, Bilbao: Elisa Garrote

[15]Hospital Insular Materno Infantil, Las Palmas de Gran Canaria: Elena Colino

[16]Hospital Virgen de la Candelaria, Santa Cruz de Tenerife: Jorge Gómez Sirvent

[17]Hospital de Lanzarote: Mónica Garzón, Vicente

[18]Complejo Universitario de Canarias, La Laguna-Tenerife: Abián Montesdeoca, Mercedes Mateo

[19]Hospital de Poniente, El Ejido: María José Muñoz, Raquel Angulo

[20]Hospital Virgen del Rocio, Sevilla: Olaf Neth, Lola Falcón

[21]Hospital Virgen de la Macarena, Sevilla: Pedro Terol

[22]Hospital Virgen de las Nieves, Granada: Juan Luis Santos

[23]Hospital Carlos Haya, Málaga: David Moreno, Esmeralda Núñez

[24]Hospital de Torrecárdenas, Almería: Francisco Lendínez

[25]Complejo Hospitalario Universitario Infanta Cristina, Badajoz: Ana Grande

[26]Complejo Hospitalario de Cáceres: Francisco José Romero

[27]Hospital de Cabueñes, Gijón: Carlos Pérez

[28]Hospital de Albacete: Miguel Lillo

[29]Hospital Virgen de la Salud, Toledo: Begoña Losada

[30]Hospital Virgen del Camino, Pamplona: Mercedes Herranz

[31]Hospital Miguel Servet, Zaragoza: Matilde Bustillo, Carmelo Guerrero

[32]Hospital Clínico Lozano Blesa, Zaragoza: Pilar Collado

[33]Complejo Hospitalario de Pontevedra: José Antonio Couceiro

[34]Hospital La Fe, Valencia: Amparo Pérez, Ana Isabel Piqueras, Rafael Bretón, Inmaculada Segarra

[35]Hospital San Juan de Alicante: César Gavilán

[36]Hospital Clínico de Valencia: Enrique Jareño

[37]Hospital General de Valencia: Elena Montesinos

[38]Hospital de Castellón: Marta Dapena

[39]Hospital Marqués de Valdecilla, Santander: Cristina Álvarez

[40]Hospital de León: Ana Gloria Andrés

[41]Hospital de Zamora: Víctor Marugán, Carlos Ochoa

[42]Hospital Virgen de la Arrixaca, Murcia: Santiago Alfayate, Ana Isabel Menasalvas

[43]Complejo Hospitalario San Millán-San Pedro, Logroño: Elisa de Miguel

[44]Paediatric HIV-BioBank integrated in the Spanish AIDS Research Network and collaborating. Executive committee: Maria Isabel González-Tomé, Ton Noguera, María José Mellado, Pere Soler, Claudia Fortuny, Africa Holguin, Mº Ángeles Muñoz, Pablo Rojo, Olaf Neth.

The Madrid cohort of HIV-infected children and adolescents, ascribed to the cohort of the Spanish Paediatric HIV Network (CoRISpe), is one of largest paediatric HIV-infected cohorts in Europe with 561 patients registered since the beginning of the epidemic until Dec 2015, all of them with available follow-up epidemiological-clinical-virological information. It includes almost all infected children and adolescents infected since the beginning of HIV epidemic and born since 1981, which are under clinical follow-up in nine public hospitals in Madrid. This currently represents 48% (561/1165) of the HIV-1B infected children and adolescents in Spain. By March 2016, there were 35 (6.2%) patients HIV-1/HCV coinfected in the Madrid cohort, and 15 (46.8%) of them had available HIV-1 *pol* sequences. From this cohort, a total of 71 patients were enrolled in this pilot study: 15 HIV-1B/HCV coinfected and 56 HIV-1B monoinfected pediatric patients. **Table S1** summarizes the clinical, epidemiological and virological features of the study population. At sampling time, both groups presented similar HIV-1 viremia, immunological profile, time without ART until HIV diagnosis, time under antiretroviral therapy (ART), and antiretroviral (ARV) experience to nucleoside reverse transcriptase inhibitors (NRTI) or protease inhibitors (PI). In contrast, non-nucleoside reverse transcriptase inhibitors (NNRTI) experience was lower in HIV-1B/HCV coinfected than monoinfected patients. Time under monotherapy, under combined ART (cART) or under high-active ART (HAART) was also similar across groups (**Table S1**).

Table S1: Clinical-virological features of study population at sampling time.

	HIV-1B/HCV Coinfected n (%)	HIV-1B Monoinfected n (%)	*p*-value
**Demographics**			
Number of patients	15	56	
Female gender	7 (46.7)	31 (55.4)	0.549
Age (median, [IQR])	11.03 [12.52-4.35]	10.86 [14.34-7.35]	0.923
Birth date			
1984-1992	6 (40)	28 (50)	1.00
1993-2001	9 (60)	28 (50)	
**HIV-1 transmission**			
Perinatal	14 (93.3)	55 (98.2)	0.380
Blood transfusion	1 (6.7)	1 (1.8)	
**HIV-1 diagnosis year**			
1986-1995	12 (80)	25 (52.1)	0.372
1996-2005	3 (20)	23 (47.9)	
**Time under HIV-1 infection(y, [IQR])**	11.3 [9.8-13.1]	10.9 [7.5-21.8]	0.773
**Year of first sequence**			
1993-1999	5 (33.3)	20 (35.7)	1.00
2000-2004	5 (33.3)	34 (60.7)	0.323
2005-2009	5 (33.3)	2 (3.6)	0.140
**Virologic features**			
Viral load (median RNA-HIV-1 cp/mL, [IQR])	5,820 [319-35820]	3,700 [200-52160]	0.346
Years under HIV exposure (mean)	11.26 ± 4.85	11.06 ± 4.99	0.641
**Immunologic status**			
CD4+ T cells % (median, [IQR])	26 [20.31-37]	32 [23.75-38.08]	0.375
CD4+ T cells mm3 (median, [IQR])	665 [504.5-1159]	719 [559.2-821]	0.941
CD8+ T cells % (median, [IQR])	42 [34-51]	38 [30.50-49.50]	0.234
CD8+ T cells mm3 (median, [IQR])	1,077 [460, 2639]	970 [50, 2754]	0.172
**ART status**			
Drug-naïve	1 (6.7)	8 (14.3)	1.00
Under ART	14 (93.3)	48 (85.7)	
**Years under ART**			
<5 years	5 (35.7)	23 (41.8)	
≥5 years	9 (64.3)	32 (58.2)	0.677
Unknown	0	1 (2.1)	
**Ever without ART**[D]			
No	0	0	1.000
Yes	15 (100)	56 (100)	
**Time without ART**[D]			
Months (median,[IQR])	34 [5.4-70.5]	37.6 [5.8-62.5]	0.822

	HIV-1B/HCV Coinfected n (%)	HIV-1B Monoinfected n (%)	*p*-value
**Ever under monotherapy[D]**			
No	4 (26.7)	31 (55.4)	0.051
Yes	11 (73.3)	25 (44.6)	
**Time under monotherapy**			
Months (medians[IQR])	31.9 [15.5-47.3]	20.4 [9.3-42.6]	0.525
**Ever under cART**			
No	4 (26.7)	23 (41.1)	0.307
Yes	11 (73.3)	33 (58.9)	
**Time under cART**			
Months (medians[IQR])	8.7 [4.9-10.8]	8.2 [4.4-17.2]	0.674
**Ever with HAART**			
No	10 (66.7)	44 (78.6)	0.331
Yes	5 (33.3)	12 (21.4)	
**Time on HAART**			
Months (medians[IQR])	65.4 [47.8-97.8]	49.8 [8.8-78.9]	0.191
**ARV experience**			
**NRTIs**	**12/14 (85.7)**	**36/37 (97.3)**	0.892
AZT	12 (100)	30 (83.3)	0.553
3TC	8 (66.67)	28 (77.8)	0.300
DDI	11 (91.67)	30 (83.3)	0.553
D4T	11 (91.67)	31 (86.1)	0.584
ABC	2 (16.67)	9 (25)	1.00
TDF	0 (0)	2 (5.6)	1.00
ddC	0	1 (2.8)	1.00
**NNRTIs**	**3/13 (23.1)**	**22/37 (59.5)**	**0.024**
EFV	3 (100)	17 (77.3)	0.331
NVP	0 (0)	9 (40.9)	0.588
RPV	0	1 (4.5)	0.647
**PIs**	**10/13 (76.9)**	**41/48 (14.6)**	0.112
LPV/r	2 (20)	9 (21.9)	1.00
NFV	7 (70)	20 (48.8)	0.550
IDV	0	9 (21.9)	0.450
SQV	1(10)	9 (21.9)	0.594
**Available HIV-1 *pol* sequences**			
PR	15 (100)*	56 (100)	1.00
RT	13 (86.7)	46 (82.1)	0.963
PR sequences from treated patients	14 (93.3)	48 (85.7)	0.955
RT sequences from treated patients	13 (92.9)	37 (77.1)	0.647

Significance threshold: *P*-value<0.05; IQR, interquartile range; ART, antiretroviral therapy; ARV, antiretroviral; DRM, drug resistance mutation; NRTI, nucleoside reverse transcriptase

inhibitors; NNRTI, non-nucleoside reverse transcriptase inhibitors; PI, protease inhibitors. ATV, atazanavir; DRV, darunavir; FPV, fosamprenavir; IDV, indinavir; NFV, nelfinavir; SQV, saquinavir; 3TC, lamivudine; ABC, abacavir; AZT, zidovudine; d4T, estavudine; ddI, didanosine; FTC, emtricitabine; TDF, tenofovir; EFV, efavirenz; ETV, etravirine; NVP, nevirapine; RPV, rilpivirine. PR, HIV-1B protease; RT, HIV-1B retrotranscriptase; D: until HIV diagnosis; cART: combined antiretroviral therapy (two combined drugs); HAART: highly active ART (two combined family drugs in one regimen). * One record from coinfected patients was a resistance profile. Regarding PR, we only analyzed 285 of the 297 aa sites of HIV-1B PR sequences, as they were available in most coinfected patients under study.

Table S2. Genbank accession numbers from HIV-1 in the study groups.

Pediatric Group	HIV-1BPR GenBank accession numbers
**HIV-1B/HCV coinfected (n= 14)**	HQ426719, HQ426726, HQ426728, HQ426733, HQ426734, HQ426781, HQ426807, HQ426818, HQ426838, HQ426843, HQ426858, HQ426863, KP881489 and KP881494
**HIV-1B monoinfected (n= 54)**	HQ426717, HQ426720, HQ426722, HQ426727, HQ426737, HQ426748, HQ426749, HQ426752, HQ426758-HQ426763, HQ426770, HQ426783, HQ426784, HQ426790, HQ426800, HQ426806, HQ426809, HQ426814, HQ426815, HQ426819-HQ426821, HQ426823, HQ426825, HQ426826, HQ426840, HQ426844, HQ426849, HQ426850, HQ426854, HQ426856, HQ426861, HQ426866, HQ426871, HQ426878, HQ426884, HQ426886, HQ426891, JQ351956, JQ351961, JQ351967, JQ351989, JQ351992, JQ351997, JQ352003, KP881484, KP881488, KP881492, KX607058, KX607067, KX607070, KX607077.

Table S3. Protease inhibitor experience and resistance mutations at HIV-1B protease.

	**HIV-1B/HCV Coinfected N=15 (%)**	**HIV-1B Monoinfected N=56 (%)**	***p*-value**
**Experience to PI**	10/14 (71.4)	41/48 (14.6)	0.112
LPV/r	2 (20)	9 (21.9)	1.00
NFV	7 (70)	20 (48.8)	0.550
IDV	0	9 (21.9)	0.450
SQV	1(10)	9 (21.9)	0.594
**Prevalence of DRM to PI**			
	5/15(33.3)	28/56(50%)	0.065
**DRM to PI (major)**			
D30N	4(80)	11(39.3)	0.640
M46I	0	11(39.3)	0.144
Q58E	0	1(3.6)	1.00
T74P	0	1(3.6)	1.00
L76V	0	1(3.6)	1.00
V82A	1(20)	7(25)	1.00
I84V	0	1(3.6)	1.00
N88S	0	2(7.1)	0.931
L90M	0	2(7.1)	0.931

PI, protease inhibitor; DRM, drug resistance mutations.

Table S4. Results from ten bootstrapped replicates (n=11) from 41 sequences of HIV monoinfected group.

	Genetic distances		Non-synonymous mutations		Synonymous mutations		Selection Pressure	
	1. π	Comparison HIV/HCV	d_N	Comparison HIV/HCV	d_S	Comparison HIV/HCV	d_N- d_S	Comparison HIV/HCV
HIV+/HCV+ (n=11)	0.052±0.01	-	0.045±0.01	-	0.074±0.03	-	-0.026±0.018	-
HIV+/HCV- (n=41)	[0.035,0.055]	NS	0.025±0.01	*	[0.038,0.118]	NS	[-0.066,-0.04]	*
HIV+/HCV- Rep.1	[0.046,0.064]	NS	[0.011,0.031]	*	[0.037,0.077]	NS	[-0.06,-0.02]	*
HIV+/HCV- Rep.2	[0.041,0.059]	NS	[0.015,0.035]	*	[0.047,0.091]	NS	[-0.024, 0.022]	*
HIV+/HCV- Rep.3	[0.05,0.066]	NS	[0.013,0.022]	*	[0.035,0.079]	NS	[-0.021,0.031]	*
HIV+/HCV- Rep.4	[0.051,0.069]	NS	[0.015,0.035]	*	[0.037,0.117]	NS	[-0.068,-0.048]	*
HIV+/HCV- Rep.5	[0.069,0.089]	*	[0.061,0.087]	*$	[0.053,0.113]	NS	[-0.032, 0.014]	NS
HIV+/HCV- Rep.6	[0.041,0.059]	NS	[0.014,0.036]	*	[0.047,0.091]	NS	[-0.022, 0.024]	*
HIV+/HCV- Rep.7	[0.053,0.071]	NS	[0.019,0.038]	*	[0.041,0.107]	NS	[-0.058,-0.038]	*
HIV+/HCV- Rep.8	[0.043,0.057]	NS	[0.041,0.059]	NS	[0.019,0.051]	*$	[-0.005,0.035]	*$
HIV+/HCV- Rep.9	[0.05,0.068]	NS	[0.012,0.036]	*	[0.037,0.077]	NS	[-0.071,-0.031]	*
HIV+/HCV- Rep.10	[0.04,0.054]	NS	[0.037,0.055]	NS	[0.032,0.088]	NS	[-0.044,0.018]	NS

Significance was calculated according to 95% confidence intervals. NS: not significant; *, significant; *$: significant and different from the original set. Only two replicates presented differences with the real original data.

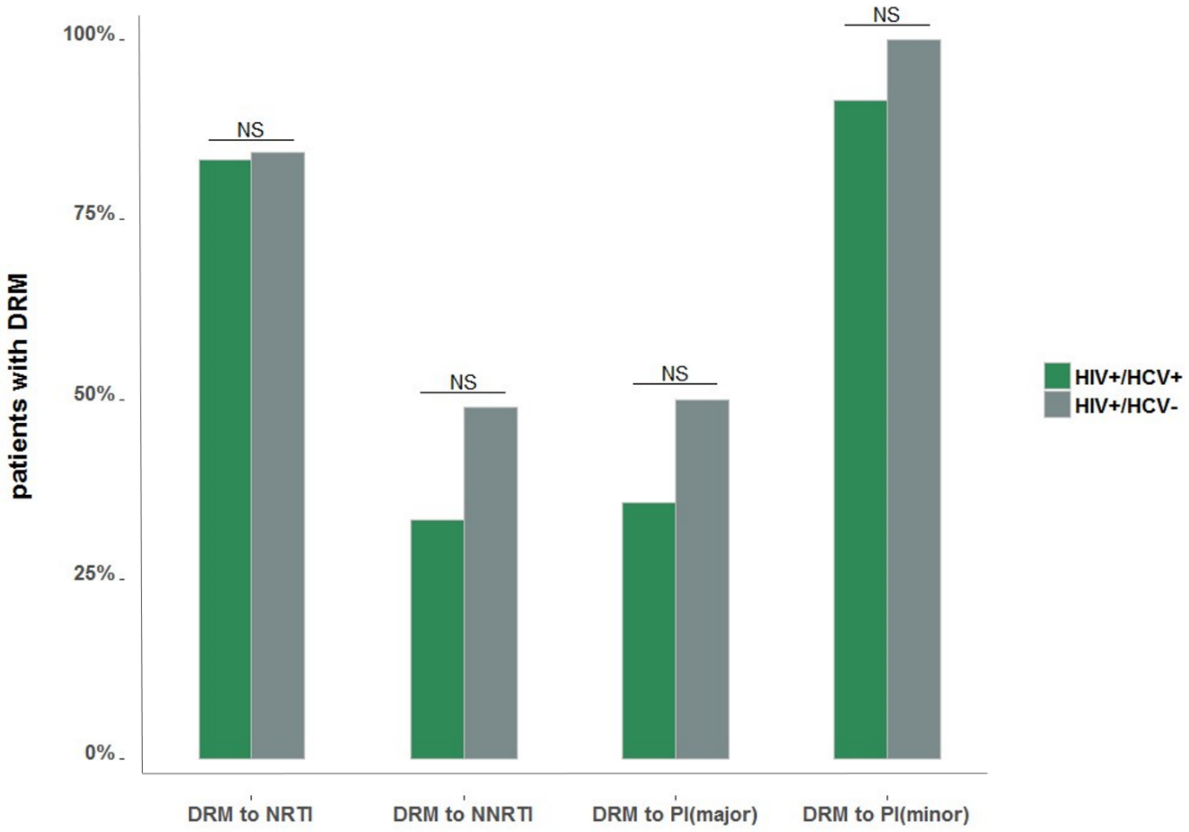

Figure S1. Prevalence of DRM to ARV families between 14 coinfected and 48 monoinfected pediatric patients under ART with available resistance information. DRM, Drug Resistance Mutation; NRTI, nucleoside reverse transcriptase inhibitors; NNRTI, non-nucleoside reverse transcriptase inhibitors; PI, protease inhibitors (major, primary mutations to PI; minor, secondary mutations to PI); NS: Not Significant *p*-value>0.05. Some DRM to NRTI at RT were more frequent among coinfected patients compared to monoinfected patients: D67N (80% *vs.* 37%; X^2=4.15, *p*=0.030), K219QE (60% *vs*. 23%, X^2=3.4; *p*-value=0.040). No differences were found in DRM to NNRTI comparing both groups, and K103N was the most prevalent substitution at RT. Among the major DRM to PI considered (30, 32, 46-48, 47, 48, 50, 53, 54, 58, 74, 76, 82, 83, 84, 88, and 90), D30N and V82A at HIV-1B PR were the only found in the coinfected group and was present in 80% of treated patients. L90M at HIV-1B PR was the most prevalent DRM to PI among the HIV-1 monoinfected group (52%), and was absent in the coinfected group.

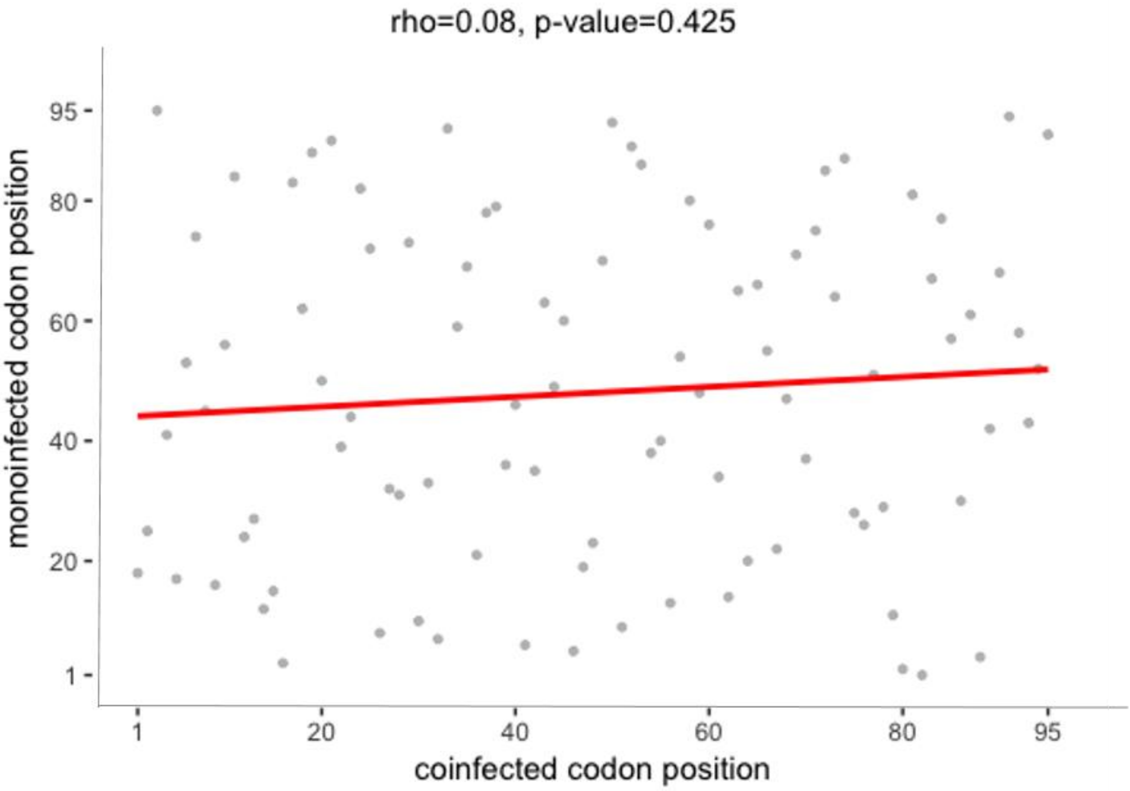

Figure S2: Spearman rank correlation of codon-specific d_N-d_S at the HIV-1BPR from monoinfected patients onto codon-specific d_N-d_S at the HIV-1BPR from coinfected patients.
